# A synergistic dual-mode biosensor for methotrexate detection leveraging FRET and Ag NP-based UV absorption

**DOI:** 10.1039/d5ra08792a

**Published:** 2026-01-30

**Authors:** Qiongdan Zhang, Qiongwei Teng, Huihong Duan, Caiyun Peng, Wenbing Sheng, Wei Wang, Bin Li

**Affiliations:** a TCM and Ethnomedicine Innovation & Development International Laboratory, School of Pharmacy, Hunan University of Chinese Medicine Changsha China wangwei402@hotmail.com libin@hnucm.edu.cn +86 15874100917 +86 13657438606

## Abstract

A novel dual-mode sensing platform (DMSP) integrating fluorescence and ultraviolet (UV) detection was developed for the sensitive and accurate quantification of methotrexate (MTX). This approach combines a FRET-based fluorescent “turn-on” mechanism, employing FAM as the fluorophore and BHQ as the quencher, with a UV absorption mode utilizing the unique surface plasmon resonance properties of silver nanoparticles (Ag NPs). The fluorescence mode enables highly sensitive detection *via* target-induced structural switching, while the AgNP-based UV channel provides a complementary, concentration-dependent signal at 405 nm, allowing cross-validation and improved reliability. The platform demonstrates a good linear detection range (0.5–20 µM), with detection limits of 0.56 µM and 0.62 µM for the fluorescence and UV modes, respectively, in serum samples. It also exhibits excellent specificity against structurally similar molecules. This dual-mode strategy offers a robust and practical alternative for therapeutic drug monitoring of MTX in complex biological environments.

## Abstract 2

A synergistic dual-mode biosensor for methotrexate detection leveraging FRET and Ag NPs based UV absorption.

## Graphical abstract

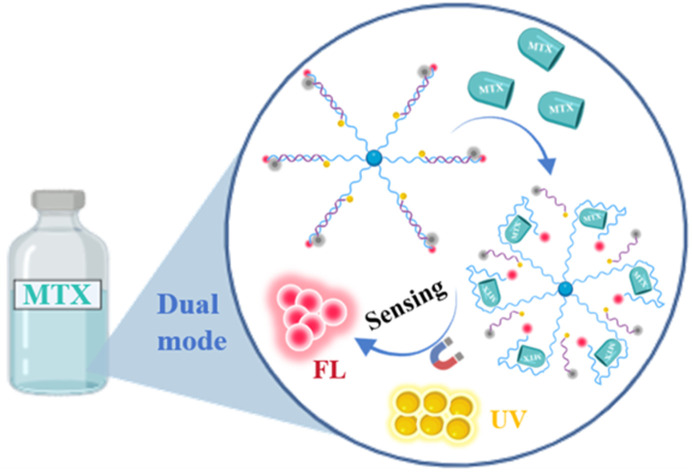

## 1. Introduction

Methotrexate (MTX) is an essential chemotherapeutic agent and immunosuppressant, used in the treatment of malignancies such as leukemia, lymphoma, and breast cancer, as well as autoimmune diseases including rheumatoid arthritis and psoriasis.^1–6^ Due to its narrow therapeutic window, maintaining MTX concentrations within the optimal range is critical to avoid serious adverse effects such as hepatotoxicity, bone marrow suppression, and renal impairment, highlighting the importance of accurate therapeutic drug monitoring (TDM).^7–9^ Conventional methods for MTX quantification-mainly high-performance liquid chromatography (HPLC) and liquid chromatography-mass spectrometry (LC-MS)-are associated with significant practical limitations.^10–13^ Although HPLC offers high accuracy, it is technically demanding, time-consuming, and requires costly instrumentation. While enzyme-linked immunosorbent assay (ELISA) is simpler to operate, it often lacks sufficient sensitivity at ultralow concentrations, restricting its use in rapid clinical or point-of-care settings.

Notably, even emerging single-mode sensing platforms-such as those relying solely on fluorescence or UV absorption-face inherent constraints that affect their reliability in complex biological matrices. Fluorescence-based single-mode systems, despite their high sensitivity, are often vulnerable to photobleaching, background interference from sample autofluorescence (*e.g.*, from serum proteins), or signal quenching by coexisting compounds, which can lead to false-positive signals or inaccurate quantification. Conversely, UV absorption-only methods, though less prone to photobleaching, generally exhibit lower specificity; their signals can be easily influenced by other UV-absorbing species present in biological fluids (*e.g.*, amino acids, metabolites) or by variations in the sample matrix, compromising detection accuracy. Moreover, single-mode approaches lack built-in cross-validation: signal deviations caused by matrix effects, instrumental drift, or operational errors cannot be internally verified, increasing the risk of unreliable readings in clinical TDM where precision is essential. These shortcomings underscore the need for a more integrated sensing strategy that simultaneously addresses sensitivity, specificity, and robustness.

To address these challenges, we developed a dual-mode sensing platform that combines fluorescence and ultraviolet (UV) absorption detection for accurate, sensitive, and reliable quantification of MTX. The first sensing modality employs a Förster resonance energy transfer (FRET) system using FAM as the fluorophore and BHQ as the quencher.^14–20^ In the absence of MTX, the close proximity of FAM and BHQ on a hybridized probe maintains the fluorescence in an “off” state. Upon introduction of MTX, displacement of the BHQ-labeled strand separates the FRET pair, restoring FAM emission and switching the fluorescence signal to an “on” state. This turn-on response provides a sensitive and selective fluorescent readout directly proportional to MTX concentration.

Complementing the fluorescence mode, the second detection modality utilizes the distinct UV absorption properties of silver nanoparticles (Ag NPs). Ag NPs exhibit a strong and well-defined surface plasmon resonance (SPR) peak around 405 nm,^21–24^ making them excellent optical reporters for UV-based sensing. In the presence of MTX, the released nucleotides alter the local environment of the Ag NPs, leading to a concentration-dependent increase in UV absorption intensity at this characteristic wavelength. This Ag NP-based UV signal provides an independent and robust quantitative output, enabling cross-validation with the fluorescence channel and reinforcing the overall reliability of the detection system.

Together, these two modes form an integrated dual-mode sensing platform that directly addresses the limitations of single-mode methods. The high sensitivity of the FRET-based fluorescence mode compensates for the relatively lower specificity of stand-alone UV absorption, while the matrix tolerance of the Ag NP-based UV detection alleviates issues such as background interference and photobleaching encountered in fluorescence-only systems. Crucially, the built-in cross-validation between two independent sensing mechanisms allows anomalies arising from matrix effects, instrumental variation, or procedural inconsistencies to be identified and corrected, greatly reducing the risk of erroneous results-a key advantage for clinical TDM where accuracy is paramount. By combining the benefits of FRET-mediated fluorescence turn-on and Ag NP-enhanced UV absorption, our system offers a promising alternative to conventional MTX assays and existing single-mode sensors, with potential for application in clinical therapeutic monitoring and point-of-care testing.

## 2. Methods

### 2.1 Polyacrylamide gel electrophoresis (PAGE) analysis

Oligonucleotides were diluted to 10 µM with reaction buffer, denatured at 95 °C for 5 min, and gradually cooled to 4 °C for annealing. Subsequently, 10 µL aliquots of each DNA sample (1 µM) were combined with 6× loading buffer and stained with CYBR gold nucleic acid dye. The mixtures were then resolved on a 12% polyacrylamide gel prepared with 1× TBE running buffer, using electrophoresis at 120 V for 40 min. Gel images were captured using a Tanon 5200 imaging system (Beijing, China).

### 2.2 Dynamic light scattering (DLS) measurements

Ag NPs dispersions were diluted 100-fold with ultrapure water to achieve a suitable concentration range (0.001–1 mg mL^−1^) and filtered through a 0.22 µm membrane prior to analysis. DLS measurements were performed on an NS-90Z Plus instrument (Malvern Panalytical, China) equipped with a 633 nm laser and a 90° scattering angle to ensure reliable detection of particle size distribution.

### 2.3 Analysis of biological samples

Human blood samples were obtained from the first hospital of Hunan University of Chinese Medicine (Changsha, China). To remove large vesicles and cellular debris, samples were centrifuged at 10 000×*g* for 10 min, and the resulting supernatant was analyzed using the dual-mode sensing platform.

### 2.4 Ethics statement

This study received approval from the ethics committee of the first affiliated hospital of Hunan University of Chinese Medicine (Approval No. HN-LL-KY-2024-025-01). All procedures complied with the principles of the Declaration of Helsinki and Good Clinical Practice (GCP). Written informed consent was obtained from all participants prior to sample collection. The study protocol, consent forms, and recruitment procedures were reviewed and approved by the ethics committee to ensure full adherence to ethical standards.

## 3. Results and discussion

### 3.1. Design principle

**Scheme 1:**
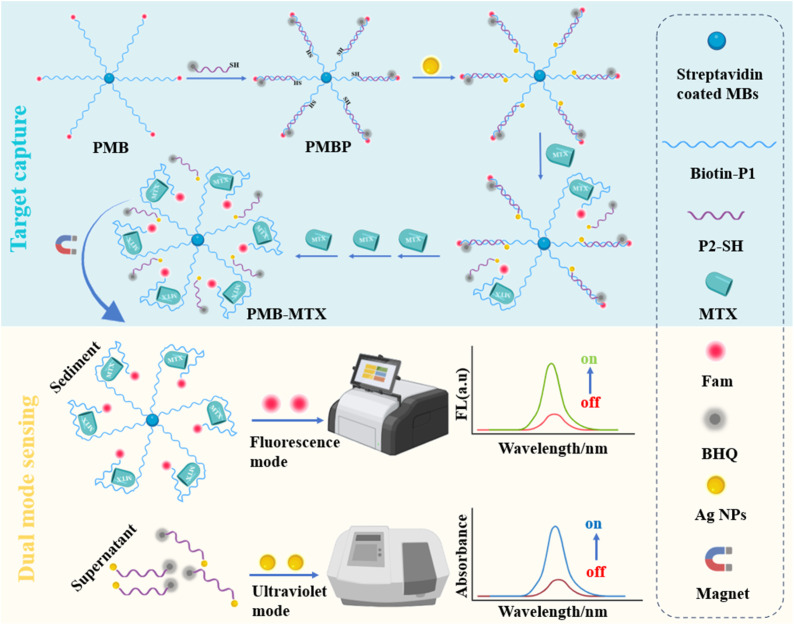
Schematic illustration of the DMSP based on the dual-mode sensing for MTX detection.

The UV and fluorescence dual-mode sensing platform (DMSP) developed for detecting methotrexate (MTX) operates through a well-defined two-step mechanism, as illustrated in Scheme 1. The process begins with target capture (blue background), where a FAM fluorophore-labeled P1 probe is immobilized on magnetic beads (PMB), and a P2 probe-modified with a BHQ quencher at its 5′-end and a thiol group (SH) at its 3′-end-hybridizes with PMB to form a duplex DNA-magnetic bead probe (PMBP). In this hybridized state, fluorescence is quenched due to Förster Resonance Energy Transfer (FRET) between FAM and BHQ, resulting in an “off” signal. In the presence of MTX, P2 is competitively displaced from the PMBP, leading to the formation of a PMB-MTX complex. After magnetic separation, FAM-labeled single-stranded nucleotides are released into the sediment, restoring a strong fluorescence signal (“on” state). Simultaneously, SH-labeled nucleotides are liberated into the supernatant, where they interact with silver nanoparticles (Ag NPs). Leveraging the UV absorption properties of Ag NPs, an increase in MTX concentration leads to enhanced UV absorption intensity (orange background). This dual-mode sensing approach enables highly sensitive and accurate quantitative detection of MTX.

### 3.2. Characterization of Ag NPs

**Fig. 1:**
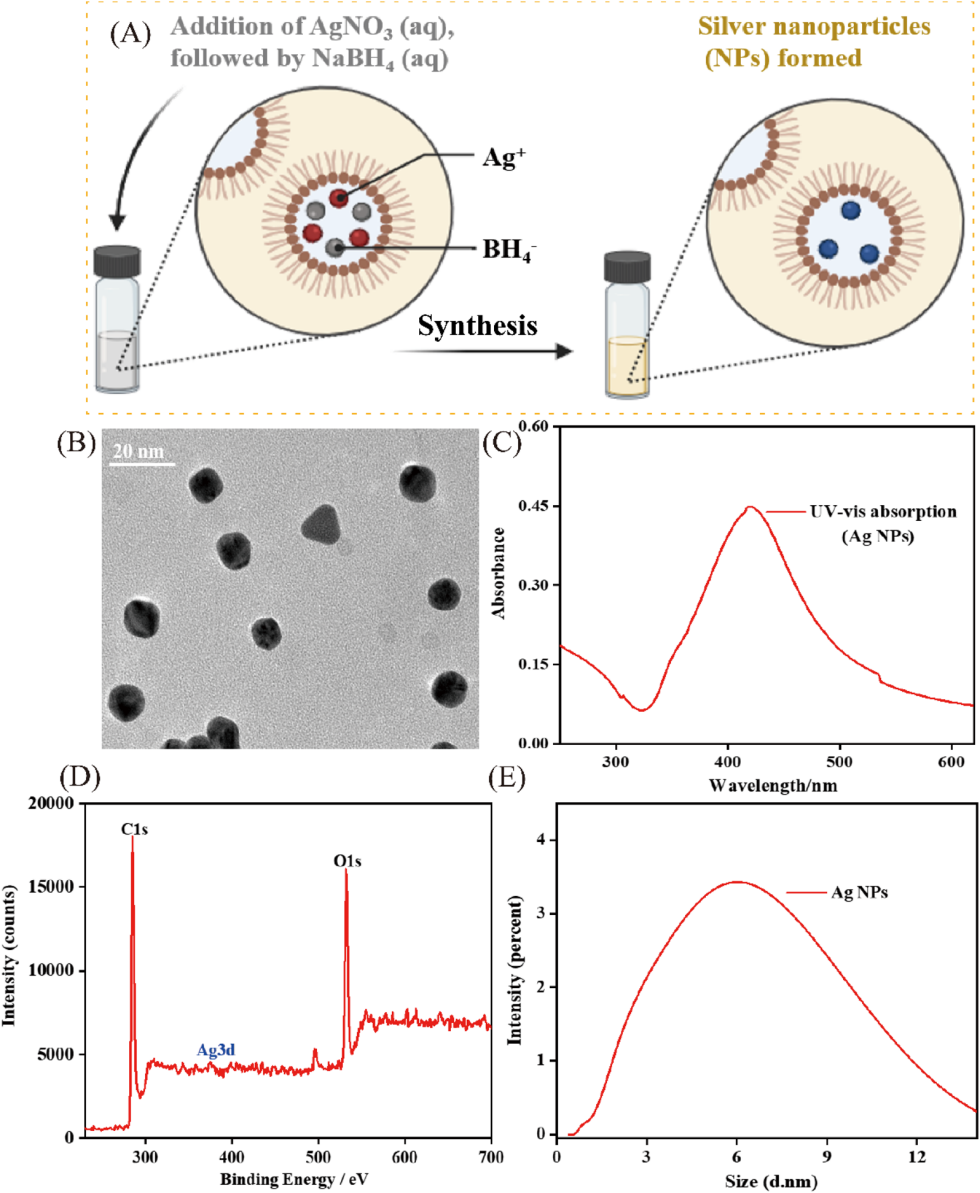
Characterization of Ag NPs. (A) Schematic illustration of the synthesis process of Ag NPs. (B) TEM images of Ag NPs. (C) UV-vis absorption spectrum of Ag NPs. XPS images (D) and DLS images (E) of Ag NPs.

The synthesis process of silver nanoparticles (Ag NPs) is shown in Fig. 1A. The morphological properties of the synthesized Ag NPs were initially analyzed using transmission electron microscopy (TEM), as depicted in Fig. 1B. The TEM image and corresponding size distribution histogram revealed spherical nanoparticles with an average diameter of approximately 7 nm, which was further corroborated by dynamic light scattering (DLS) analysis (Fig. 1E). UV-vis absorption spectroscopy was subsequently employed to confirm the formation of Ag NPs. A distinct absorption peak centered at 405 nm was observed in Fig. 1C, which is attributed to the localized surface plasmon resonance (LSPR) typical of Ag NPs. Furthermore, X-ray photoelectron spectroscopy (XPS) analysis provided additional evidence for the successful synthesis, with the Ag 3d peak appearing at 371.5 eV (Fig. 1D), confirming the presence of elemental silver in the synthesized material. The stability of the synthesized Ag NPs was also evaluated and showed promising potential for detection in complex media, as they maintained high UV-vis absorbance intensity across a range of diluted human serum concentrations (Fig. S1A). The assembly process was systematically investigated, beginning with the conjugation of Ag NPs to P2 strands. Characterization of the Ag NPs-P2 conjugates revealed an increase in particle size by DLS (Fig. S1B) and observable aggregation in TEM (Fig. S1C–S1E), confirming successful bioconjugation and indicating that target binding induces a measurable morphological change in the probe components. To comprehensively verify the construction of the final PMBP probe (magnetic bead-P1-P2-Ag NPs complex), the morphological evolution was directly visualized by TEM (Fig. S1D), showing the transition from smooth-surfaced bare magnetic beads MBs, (a) to a rougher texture after DNA strand conjugation (b), and a further increase in surface roughness and particle size following Ag NPs modification (c). This sequential growth was quantitatively corroborated by DLS, which recorded a marked increase in the average hydrodynamic diameter from approximately 800 nm for bare MBs to 880 nm for the fully assembled probe (Fig. S1H). Zeta potential measurements offered additional support for the assembly process (Fig. S1F): while bare MBs and Ag NPs controls exhibited negative charges of −8 mV and −17 mV, respectively, conjugation of DNA to MBs resulted in a slight increase in negative potential, and the final PMBP probe showed a significantly enhanced negative charge of approximately −30 mV, consistent with the cumulative contribution from the negatively charged nucleic acid backbone and Ag NPs. Elemental composition of the final architecture was confirmed by XPS survey spectra (Fig. S1G), where the distinct presence of both Ag 3d (from Ag NPs) and Fe 2p (from magnetic beads) peaks provided unambiguous evidence for the successful integration of all designed components. Finally, stability assessments underscored the probe's practical utility, demonstrating that it retained over 80% of its initial fluorescence signal across an 8-hour period (Fig. S1I) and maintained structural integrity in varying concentrations of diluted human serum (Fig. S1J), thereby confirming its suitability for biological sensing applications.

### 3.3. Feasibility analysis of DMSP

**Fig. 2:**
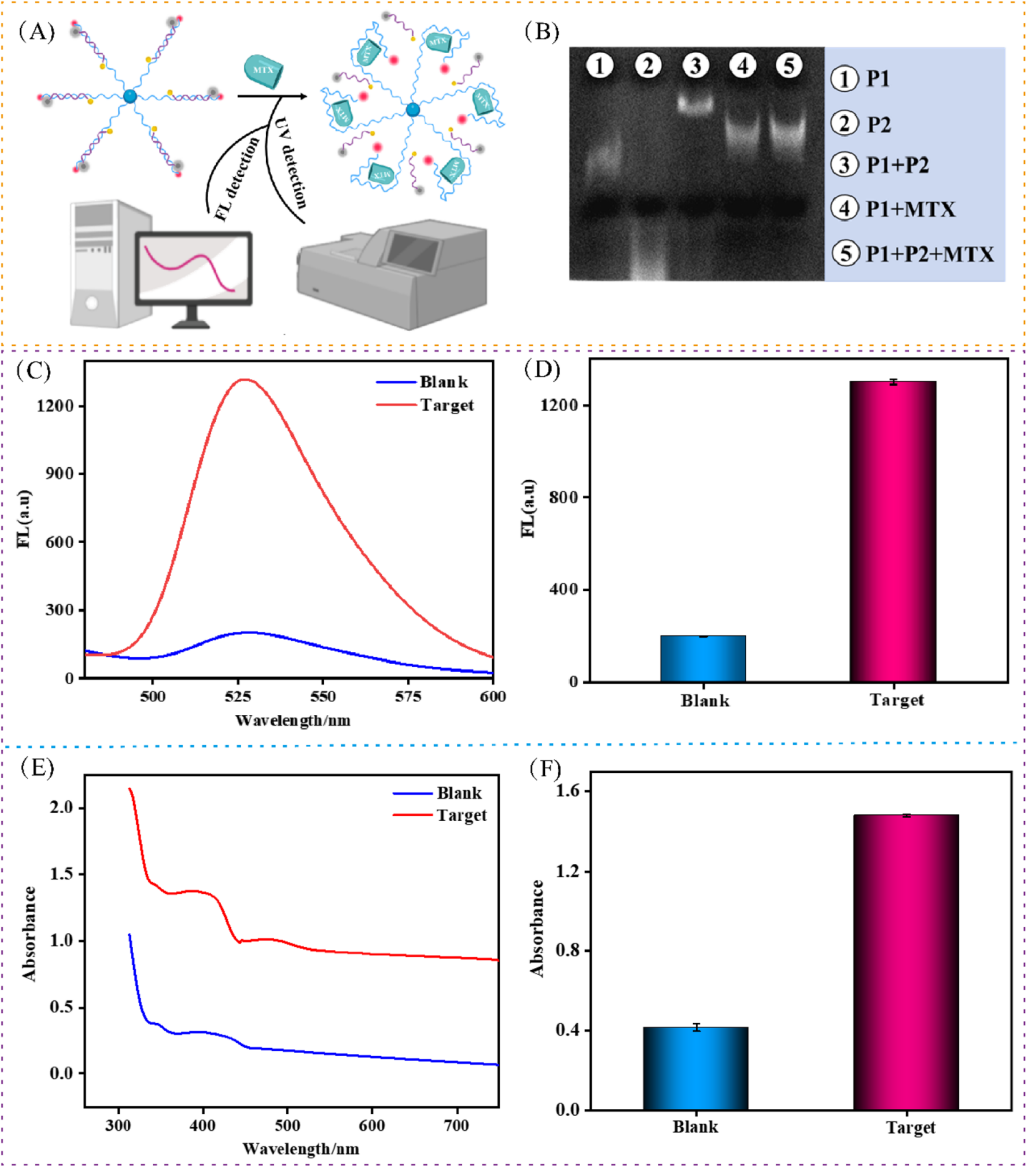
Feasibility validation of the DMSP. (A) Schematic illustration of the DMSP. (B) PAGE analysis: lane 1: P1; lane 2: P2; lane 3: P1 + P2; lane 4: P1 + MTX (50 µM); lane 5: P1 + P2 + MTX (50 µM). (C) Fluorescence emission spectra of the system in the presence (red) and absence (blue) of MTX. (D) Histogram analysis of nanosensor corresponded to the results from (C). (E) UV-vis absorption spectra of the system in the presence (red) and absence (blue) of MTX. (F) Histogram analysis of nanosensor corresponded to the results from (E). Data represent means ± SD. (*n* = 5).

Fig. 2A illustrates the schematic diagram of the ultraviolet and fluorescence (FL) dual-mode sensing strategy for MTX detection. To validate the feasibility of the sensing mechanism, polyacrylamide gel electrophoresis (PAGE) was performed. As shown in Fig. 2B, lane 1 and lane 2 correspond to the free strands P1 and P2, respectively. A distinct, stronger band observed in lane 3 confirms the formation of a stable double-stranded structure (P1-P2) through hybridization. When MTX was introduced (lane 5), a new band appeared, corresponding to the P1-MTX complex. This band aligns with that in lane 4, indicating the optimal structural transition and confirming that MTX effectively triggers the DMSP activation process.

For the FL detection mode, in the absence of the target, hybridization between P1 and P2 results in a duplex probe (P1/P2) where FAM fluorescence is quenched by BHQ, rendering the system in an “off” state. In the presence of target MTX, P2 is displaced from the duplex, leading to the formation of a MTX/P1 complex and the release of FAM-labeled fragments. This restoration of fluorescence switches the system to an “on” state (Fig. 2C and D). The fluorescence intensity at 525 nm increased progressively with higher MTX concentrations, demonstrating the high sensitivity of this method and enabling effective quantitative detection of MTX. The ultraviolet mode similarly leverages the UV-vis absorption of Ag NPs at 405 nm. Higher MTX concentrations lead to increased aggregation of the P2-modified Ag NPs, which gives rise to intensified absorption and serves as a complementary detection signal (Fig. 2E and F).

In addition, we further verified the feasibility of the key FRET mechanisms involved in the experiment. As detailed in Fig. S2, first, circular dichroism (CD) spectroscopy (Fig. S2A) was used to track target-induced DNA conformational changes. The CD spectrum of the P1-P2 duplex (“Without MTX”) displays a characteristic peak at 270 nm and 300 nm. Upon MTX addition, this spectrum undergoes a pronounced shift, converging toward the profile of “Free P1,” which provides direct evidence for the MTX-triggered dissociation of the P1-P2 duplex-the structural prerequisite for FRET disruption. Subsequently, time-resolved fluorescence lifetime measurements were conducted to directly probe the FRET activity. The average donor lifetime (Fig. S2B) significantly increased from 1.86 ns in the intact P1-P2 complex (“Without MTX,” where FRET is active and quenching occurs) to 4.23 ns after MTX addition-a value closely matching the lifetime of the free donor-labeled P1 (4.23 ns). This recovery is visually evident in the fluorescence decay curves (Fig. S2C), where the faster decay associated with the quenched donor (“Without MTX”) transitions to a slower decay kinetics upon target binding (“With MTX”). These data constitute direct, quantitative evidence that MTX binding relieves the FRET quenching.

To ensure this process is driven by specific, well-defined molecular recognition, we confirmed the binding stoichiometry between the aptamer P1 and MTX *via* fluorescence titration. As shown in Fig. S2D, the titration curve reaches a clear plateau at an MTX-to-P1 molar ratio of 1, unambiguously establishing a 1 : 1 binding model. This specificity underpins the reliability of the FRET-based sensing mechanism. Finally, the FRET process was quantitatively analyzed in terms of intermolecular distance. Based on the lifetime data, the FRET efficiency (*E*) was calculated to be 57.5% using the formula *E* = 1 − (*T*_DA_/*T*_D_). Given the Förster radius (*R*_0_) of 4.5 nm^25^ for the FAM-BHQ donor–acceptor pair, the donor–acceptor distance (*r*) in the active FRET state (“Without MTX”) was determined to be 4.22 nm *via* the relationship *r* = *R*_0_[(1 − *E*)/*E*]^1/6^. This distance is well within the *R*_0_, confirming effective energy transfer and quenching. Upon MTX addition, the inferred separation exceeds 10 nm, far beyond *R*_0_, which reduces the FRET efficiency to less than 5% and completely disrupts the energy transfer. Together, this multi-faceted analysis provides a robust and comprehensive validation of the proposed FRET-switching mechanism.

Collectively, the results from both UV-vis and FL spectroscopy confirm that the proposed dual-mode sensing platform (DMSP) is successfully constructed and exhibits efficient performance for MTX detection.

### 3.4. Optimization of experimental conditions

**Fig. 3:**
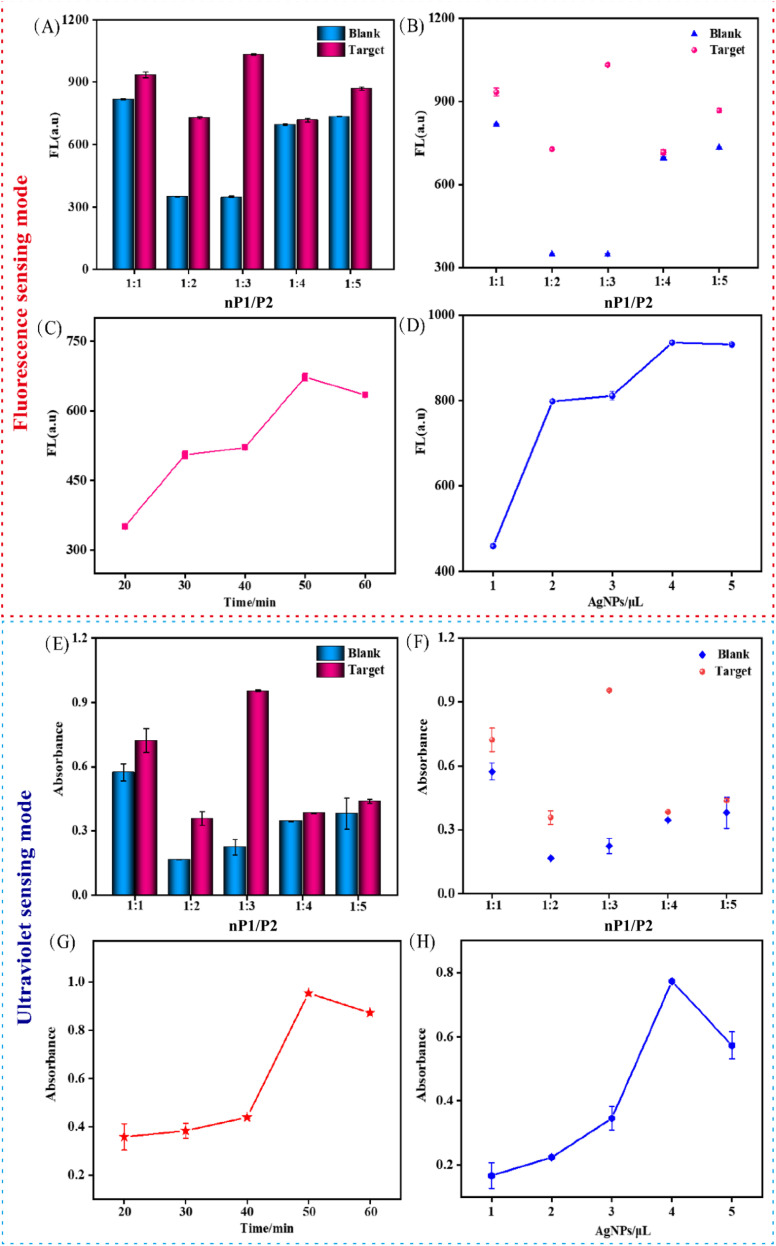
Optimization of the DMSP. (A and B) Concentration ratios of P1 and P2 by fluorescence sensing modes. (C) Hybridization time of P1 and P2 by fluorescence sensing modes. (D) Concentrations of Ag NPs by fluorescence sensing modes. (E and F) Concentration ratios of P1 and P2 by ultraviolet sensing modes. (G) Hybridization time of P1 and P2 by ultraviolet sensing modes. (H) Concentrations of Ag NPs by ultraviolet sensing modes. Data represent means ± SD. (*n* = 4).

Systematic optimization of key parameters was conducted to enhance the sensitivity of the dual-mode detection strategy. The initial optimization focused on the P1 to P2 hybridization ratio. As shown in Fig. 3A, B, E and F, a ratio of 1 : 3 was identified as optimal, yielding the greatest disparity between the fluorescence and UV-vis signals. Subsequently, the reaction time was investigated. Fig. 3C–G reveal that a duration of 50 min provided the well-defined maximum signal intensity, establishing it as the optimal reaction time. In parallel, the concentration of Ag NPs was systematically optimized to ensure optimal amplification for both detection modes, as detailed in Fig. 3D–H. A stock Ag NPs solution at a concentration of 8 mg mL^−1^ was used. Different volumes (1–5 µL) of this stock were added to a total reaction volume of 200 µL to achieve final concentrations ranging from 0.04 to 0.20 mg mL^−1^. Based on the performance evaluation, a final concentration of 0.16 mg mL^−1^, corresponding to the addition of 4 µL of the stock solution, provided effective signal amplification and was therefore selected for all subsequent experiments. This optimized condition contributes to the enhanced performance of both the fluorescence and UV detection systems.

### 3.5. Sensitive and specific dual mode detection of MTX

To systematically validate the performance of the dual-mode DMSP probe under optimized conditions, the sensing behavior was assessed sequentially in fluorescence and UV-vis absorption modes across a series of MTX concentrations (0.5–50 µM) (0, 0.1, 0.5, 1, 2, 4, 8, 10, 20, 40, 50 µM).

**Fig. 4:**
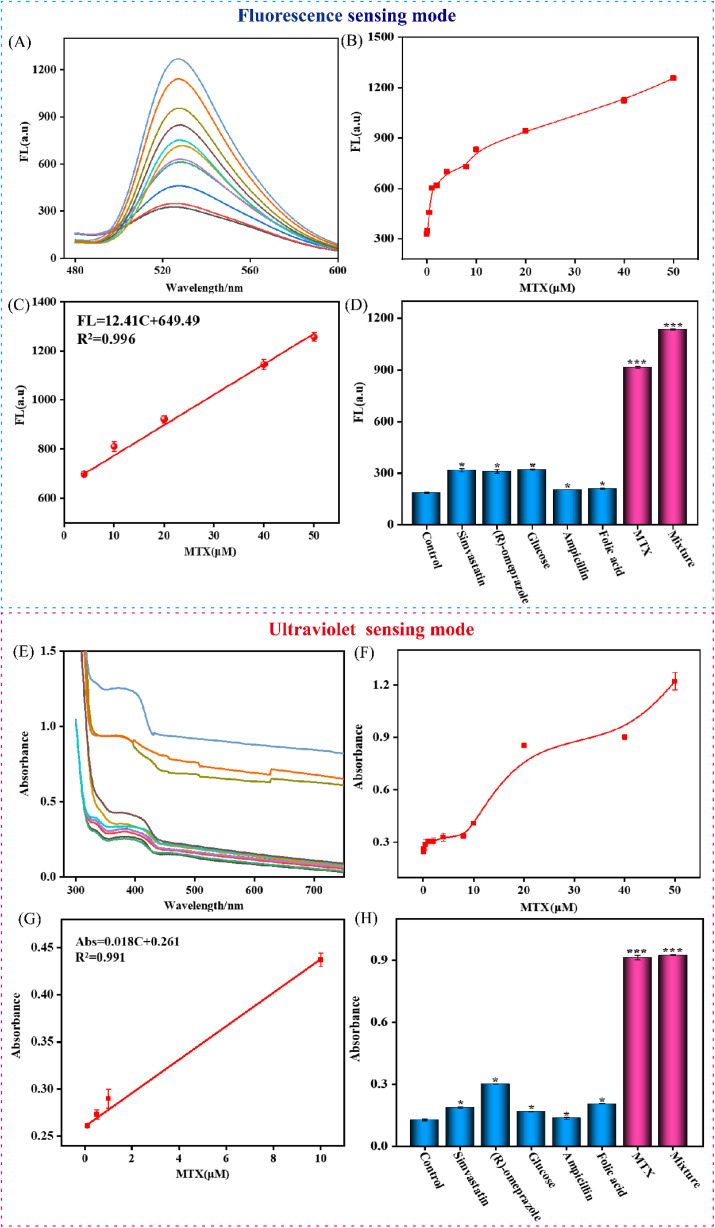
Sensitivity and selectivity of the DMSP assay. (A and B) Fluorescence intensity of different concentrations of MTX ranging from 0.5 to 50 µM (0, 0.1, 0.5, 1, 2, 4, 8, 10, 20, 40, 50 µM) by fluorescence sensing modes. (C) Linear relationship between fluorescence intensity and the concentrations of MTX by fluorescence sensing modes. (D) Selectivity for MTX by fluorescence sensing modes. (E and F) UV absorbance intensity of different concentrations of MTX ranging from 0.5 to 50 µM (0, 0.1, 0.5, 1, 2, 4, 8, 10, 20, 40, 50 µM) by UV sensing modes. (C) Linear relationship between UV absorbance intensity and the concentrations of MTX by UV sensing modes. (D) Selectivity for MTX by UV sensing modes. ***P ≤0.001 (unpaired *t*-test). Data represent means ± SD. (*n* = 4).

In the fluorescence detection mode, a notable signal-on response was observed with increasing MTX concentration, as shown in Fig. 4A and B. The fluorescence intensity exhibited a strong linear correlation with MTX concentration, fitted by the equation: FL = 12.41*C* + 649.49 (*R*^2^ = 0.996) (Fig. 4C), where *C* represents the MTX concentration. Based on this 3ó/S relationship, the detection limit (LOD) was determined to be 0.11 µM. The sensing system was subsequently evaluated under the UV-vis absorption mode for its colorimetric detection capability. As illustrated in Fig. 4E and F, the absorption intensity increased progressively with MTX concentration, enabling reliable quantitative analysis. A distinct linear calibration was established between absorbance and MTX concentration, following the equation Abs = 0.018*C* + 0.261 (*R*^2^ = 0.991), with a LOD of 0.12 µM (Fig. 4G). This result confirms the robust performance of the UV-vis mode and complements the fluorescence sensing channel.

To verify the selectivity of the dual-mode platform, both fluorescence and UV-vis responses were tested against potentially interfering substances, including Simvastatin, (*R*)-omeprazole, Glucose, Ampicillin, and Folic acid.^26^ As shown in Fig. 4D (fluorescence) and 4H (UV-vis), only MTX induced a significant signal change, while other structurally related or coexisting species produced negligible responses. Importantly, the sensor maintained high selectivity toward MTX even in the presence of a mixture of interferents. These findings collectively demonstrate that the DMSP-based dual-mode strategy exhibits excellent specificity, making it suitable for accurate MTX detection in complex matrices environments.

### 3.6. Application in detection of human serum sample

Prior to applying the PMBP probe for the detection of human serum samples, we first systematically evaluated its performance and stability in simulated biological environments. We evaluated the probe's suitability for complex biological environments (Fig. S3). Enzyme resistance was assessed by incubating the probe with DNase I across a concentration gradient (0.1–10 U per mL) and over an extended time course. The probe exhibited robust stability, retaining its fluorescence signal without significant attenuation even at the highest enzyme concentration (Fig. S3A) and maintaining >80% of its initial signal after 10 hours of incubation (Fig. S3B), indicating strong resistance to nuclease degradation. Competitive binding specificity was tested in a clinically relevant medium of diluted human serum against MTX, its structural analogs (aminopterin, pemetrexed), common serum interferents (glucose, albumin), and metal ions (Na^+^, K^+^, Ca^2+^, Mg^2+^). Both fluorescence and UV-vis sensing modes yielded consistent results: only MTX (or a mixture containing MTX) triggered a dramatic and statistically significant signal enhancement (*p* <0.001), whereas all analogs and interferents produced negligible responses (Fig. S3C and S3D). Together, these findings confirm that the PMBP probe successfully integrates a specific, MTX-driven FRET-disruption mechanism with exceptional structural stability and high selectivity. This combination of attributes validates its strong potential for reliable detection in real-world biological matrices. Subsequently, we evaluate the practical applicability of the DMSP sensor for MTX detection in complex biological media, employing a dual-mode approach (fluorescence and UV-vis absorption) in 50-fold diluted human serum. The accuracy of each mode was first verified through recovery experiments. In the fluorescence mode, recovery rates for spiked MTX (0.50–20.00 µM) ranged from 98.50% to 114.01%, with relative standard deviations (RSDs) between 0.43% and 1.21% (Table S2). Similarly, the UV-vis absorption mode yielded recoveries of 98.03–103.85% and RSDs of 0.62–1.81% (Table S5), confirming that both methods offer high accuracy in a complex matrix.

Method precision was evaluated for both the DMSP sensor (using fluorescence and UV sensing modes) and the reference HPLC method. As summarized in Tables S3 and S6, the DMSP sensor demonstrated consistent precision across all tested conditions. In the fluorescence mode, intra-day RSDs ranged from 1.20% to 3.11%, inter-day RSDs from 3.11% to 3.25%, and inter-operator RSDs from 3.23% to 4.26%. In the UV mode, intra-day RSDs were 3.25–3.45%, inter-day RSDs 4.53–5.05%, and inter-operator RSDs 4.99–5.85%. For comparison, the HPLC reference method exhibited similarly low variability: in the fluorescence-correlated assays, intra-day RSDs were ≤2.80%, inter-day RSDs ≤4.20%, and inter-operator RSDs ≤4.85%; in the UV-correlated assays, the corresponding values were ≤4.20%, ≤5.05%, and ≤4.92%, respectively (Table S4–S7).

All RSD values for both the DMSP sensor and the HPLC method fall within widely accepted bioanalytical limits, confirming that the dual-mode DMSP sensor possesses repeatability and reproducibility comparable to those of the gold-standard technique.

To validate the detection accuracy of the DMSP method, we performed a direct comparative analysis with HPLC (Fig. S4). The correlation plot (Fig. S4A) gave a linear regression equation of *Y* = 1.012*C* + 0.085 (*r* = 0.981), indicating a strong positive linear relationship between the two techniques. A Bland–Altman plot (Fig. S4B) further confirmed the agreement, showing 95% limits of agreement (LOA) of ± 0.092 nM and an overall bias of 0.15 nM; the majority of data points fell within the LOA, confirming acceptable deviation between the methods. Collectively, the recovery, precision, and correlation results consistently demonstrate that the DMSP method exhibits high concordance with the gold-standard HPLC technique, validating its reliability for accurate MTX quantification in complex biological matrices.

**Fig. 5:**
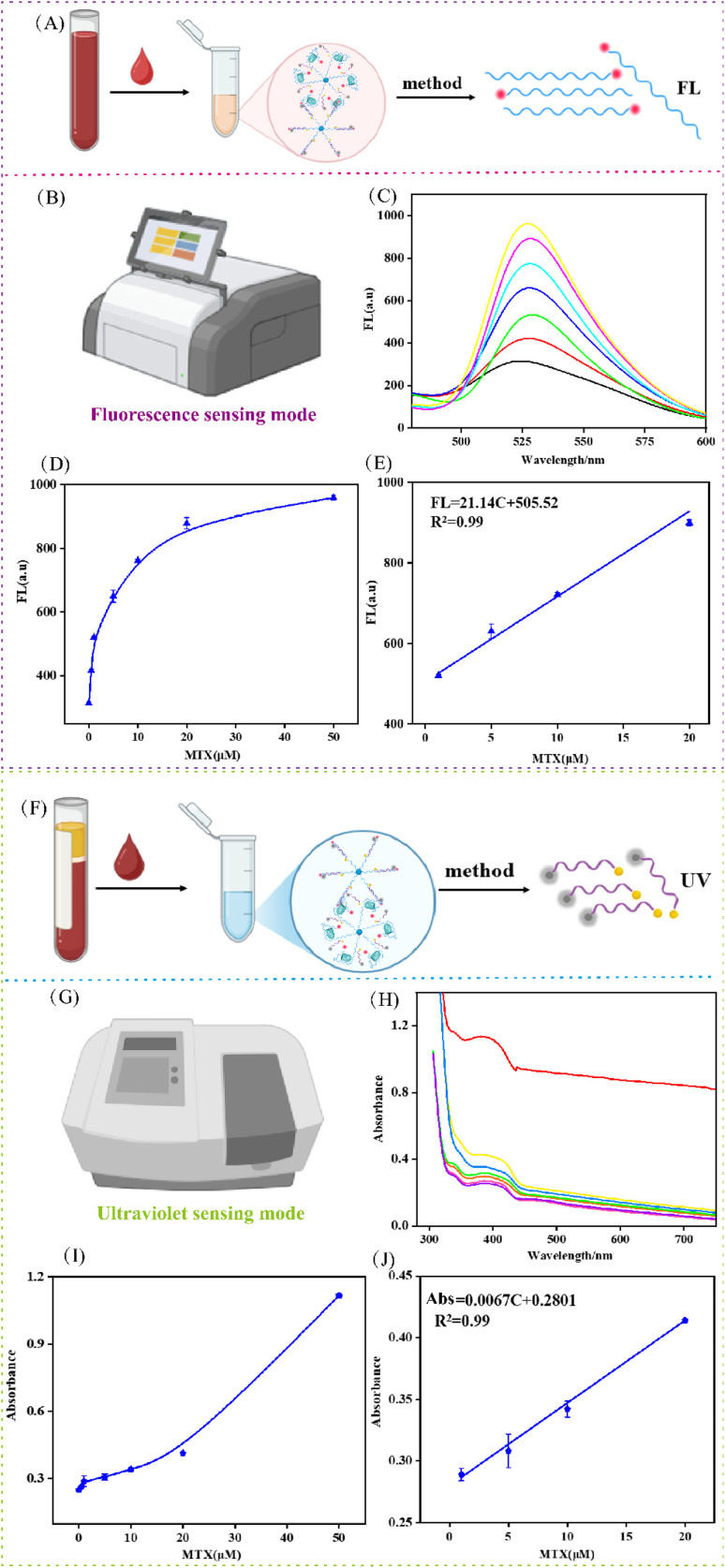
Performance analysis in biological samples. (A) Schematic representation of the processing procedure for human serum samples by fluorescence sensing modes. (B) Fluorescence signal output mode based on DMSP strategy. (C and D) Fluorescence intensity of different concentrations of MTX ranging from 0.5 to 50 µM (0, 0.5, 1, 5, 10, 20, 50 µM) in serum samples by fluorescence sensing modes. (E) Linear dependence of FL signal values by different concentrations of MTX by fluorescence sensing modes. (F) Schematic representation of the processing procedure for human serum samples by UV sensing modes. (G) UV absorbance signal output mode based on DMSP strategy. (H and I) UV absorbance intensity of different concentrations of MTX ranging from 0.5 to 50 µM (0, 0.5, 1, 5, 10, 20, 50 µM) in serum samples by UV sensing modes. (J) Linear dependence of UV signal values by different concentrations of MTX by UV sensing modes. Data represent means ± SD. (*n* = 3).

Regarding sensitivity, the fluorescence mode exhibited a strong linear correlation (FL = 21.14*C* + 505.52, *R*^2^ = 0.99) within 0.5–50 µM (0, 0.5, 1, 5, 10, 20, 50 µM) (Fig. 5B–D), achieving a detection limit (LOD) of 0.56 µM (Fig. 5E). Concurrently, the UV-vis absorption mode also showed a concentration-dependent response (Fig. 5G–I), following the equation Abs = 0.0067*C* + 0.2801 (*R*^2^ = 0.99), with a LOD of 0.62 µM (Fig. 5J).

These results demonstrate that both sensing modalities offer excellent reproducibility, accuracy, and sensitivity in complex samples. The consistency between the two modes highlights the robustness and reliability of the DMSP strategy for the precise monitoring of MTX in real biological systems.

## 4. Conclusion

In summary, we have successfully constructed a novel dual-mode sensing platform for the sensitive and reliable detection of methotrexate (MTX). The key advantage of this strategy lies in the synergistic integration of a fluorescence “turn-on” mechanism and an Ag NPs-based UV absorption signal. This design provides built-in cross-validation, where the highly sensitive FRET-based fluorescence channel and the robust, concentration-dependent UV channel mutually reinforce the analytical outcome. Consequently, the dual-signal cross-validation capability of the platform contributes to reducing the risk of false-positive readings and improves measurement reliability in complex biological matrices such as human serum. By overcoming the limitations of single-mode detection, our work demonstrates a versatile and robust approach for therapeutic drug monitoring, holding significant promise for precise clinical diagnostics and point-of-care testing applications.

## Conflicts of interest

The authors declare that they have no known competing financial interests or personal relationships that could have appeared to influence the work reported in this paper.

## Supplementary Material

RA-016-D5RA08792A-s001

## Data Availability

The datasets generated during and/or analysed during the current study are available from the corresponding author on reasonable request. Supplementary information (SI) is available. See DOI: https://doi.org/10.1039/d5ra08792a.
