# Supplementary material for: A synergistic dual-mode biosensor for methotrexate detection leveraging FRET and Ag NP-based UV absorption
Source: RSC Adv. 2026 Jan 30;16(7):6454–63. doi: 10.1039/d5ra08792a (PMC12856982; doi:10.1039/d5ra08792a)
Supplement: RA-016-D5RA08792A-s001 [file RA-016-D5RA08792A-s001.pdf]

Supplementary Information (SI) for RSC Advances.
This journal is © The Royal Society of Chemistry 2026

Supplementary information

A Synergistic Dual-Mode Biosensor for Methotrexate Detection Leveraging FRET and Ag NPs-Based UV Absorption

Qiongdan Zhang[a], Qiongwei Teng[a], Huihong Duan[a], Caiyun Peng[a], Wenbing Sheng[a], Wei Wang[a]*, Bin Li[a]*.

[a]TCM and Ethnomedicine Innovation & Development International Laboratory, School of Pharmacy, Hunan University of Chinese Medicine, Changsha, China;

Co-author: Qiongdan Zhang, Qiongwei Teng

Correspondence: Bin Li, Wei Wang

E-mail addresses: libin@hnucm.edu.cn (B.L.); wangwei402@hotmail.com (W.W.).

Materials and Reagents

All oligonucleotides (sequences provided in Table S1) were synthesized and purified by Hippo Biotechnology Co., Ltd. (Zhejiang, China). Methotrexate (MTX), Ampicillin, (R)-Omeprazole, Simvastatin, Glucose, Aminopterin, Pemetrexed, and Folic acid were supplied by Yuanye Biotechnology Co., Ltd. (Shanghai, China). $AgNO_3$, NaCl, KBH_4, HCl, NaOH, $MgCl_2$, MOPS, and phosphate-buffered saline (PBS; 137 mM NaCl, 10 mM phosphate, 2.7 mM KCl, pH 7.4) were obtained from Sigma-Aldrich (St. Louis, MO, USA). All working solutions were prepared in MOPS buffer (10 mM MOPS, 100 mM NaCl, 2.5 mM $MgCl_2$, pH 7.6). All reagents were of analytical grade or higher and stored at 4 °C prior to use.

Instrument

UV-vis absorption and fluorescence spectra were acquired using a Shimadzu UV-1800 spectrophotometer and a Hitachi FL-2500 fluorescence photometer, respectively. Ag NPs were characterized by high-resolution transmission electron microscopy (HR-TEM, JEOL Co., Japan) at 200 kV. X-ray photoelectron spectroscopy (XPS) analysis was performed on an AXIS SUPRA+ spectrometer (Shimadzu, UK). Circular Dichroism (CD) Spectroscopy analysis was performed on a J-810 CD spectrometer (JASCO, Japan). Particle size distribution was determined using a dynamic light scattering (DLS) instrument (NS-90Z Plus, Malvern Panalytical, China). Polyacrylamide gel images were captured with a Tanon 5200 dual-color fluorescence imaging system (Beijing, China). Sample incubation was conducted in an MB-102 constant-temperature metal bath shaker (Hangzhou, China).

Synthesis of Ag NPs

Ag NPs were synthesized according to a previously reported procedure with minor modifications [1]. Prior to synthesis, all glassware was thoroughly cleaned with aqua regia (3:1 HCl/HNO_3) and rinsed extensively with Milli-Q water. In brief, 500 mL of an aqueous solution containing 0.25 mM $AgNO_3$ and 0.25 mM trisodium citrate was vigorously stirred, followed by rapid addition of 1 mL of freshly prepared 0.5% $NaBH_4$. The reaction proceeded under stirring for 30 min, during which the solution turned yellow, indicating Ag NPs formation. The resulting colloid was filtered through a 0.22 μm membrane and stored at 4 °C.

P1 Strand Information

The P1 strand used in this study corresponds to the HMX38 aptamer sequence reported by He et al. [2], which was selected via a library-immobilized capillary systematic evolution of ligands by exponential enrichment (C-SELEX) strategy and exhibits specific binding affinity toward MTX.

Preparation of the PMB Complex

The PMB complex was formed by incubating 30 μL of streptavidin-coated magnetic beads (10 mg/mL) with 30 μL of biotinylated P1 (50 μM) in 200 μL of MOPS buffer under gentle shaking at room temperature for 30 min. The beads were subsequently washed three times under magnetic separation to remove unbound strands and resuspended in 300 μL of MOPS buffer. The final complex was stored at 4 °C until further use.

Dual-Mode Sensing Analysis of MTX

First, 10 μL of streptavidin-coated magnetic beads (5 mg/mL) was mixed with 10 μL of biotinylated P1 (10 μM) in 100 μL of MOPS buffer. The mixture was incubated at room temperature under gentle shaking for 30 min to form the PMB complex. After magnetic separation, unbound P1 was removed, and the beads were washed three times. The resulting complex was resuspended in 200 μL of MOPS buffer and stored at 4 °C for further use. Subsequently, 10 μL of 30 μM P2 was

added to the PMB complex and incubated at 37 °C for 50 min. Then, 4 μL of 5 mg/mL Ag NPs was introduced and further incubated at 25 °C in the dark for 45 min to facilitate Ag-S bond formation between Ag NPs and the P2 strand, yielding the PMBP complex. After adding varying concentrations of MTX to the PMBP solution and incubating at 37 °C for 50 min, magnetic separation was performed. The supernatant containing P2-Ag NPs and the sediment were collected separately. Finally, 200 μL of the sediment and the supernatant was analyzed using a fluorometer (excitation: 460 nm; emission: 480-600 nm; slit widths: 5 nm for both excitation and emission) along with UV absorption measurements, respectively.

Selectivity Assessment

To evaluate the selectivity of the sensing platform, 5 μL of each potential interferent-including 100 μM Ampicillin, (R)-Omeprazole, Simvastatin, Glucose, Folic acid, and MTX, as well as a mixture containing all these compounds-was separately added to individual PMBP complex systems. Each mixture was incubated at 37 °C for 50 min. All subsequent sample preparation and detection steps were consistent with the dual-mode sensing procedure described above.

Fluorescence Lifetime & FRET Parameter Measurement

Fluorescence lifetime measurements and FRET parameter determination were performed using a Fluorolog-3 fluorescence spectrometer (Horiba, Japan). To specifically probe the FRET process, the donor fluorophore (FAM) was excited at 490 nm, and its emission was monitored at 520 nm. The fluorescence decay curves were recorded in time-correlated single photon counting (TCSPC) mode. These decay profiles provided the direct data for calculating the donor lifetime in both the presence and absence of the acceptor, which is essential for determining FRET efficiency and the subsequent donor-acceptor distance.

Table S1 DNA sequences (5′-3′) utilized in this work

Strand	Sequence (5′-3′)
P1	Biotin-CTCTCGGGCGAACGCGGGATGTTTGGGGGACCCACGTTCGCCC-FAM
P2	BHQ-CGTTCGCCCGAGAG-SH

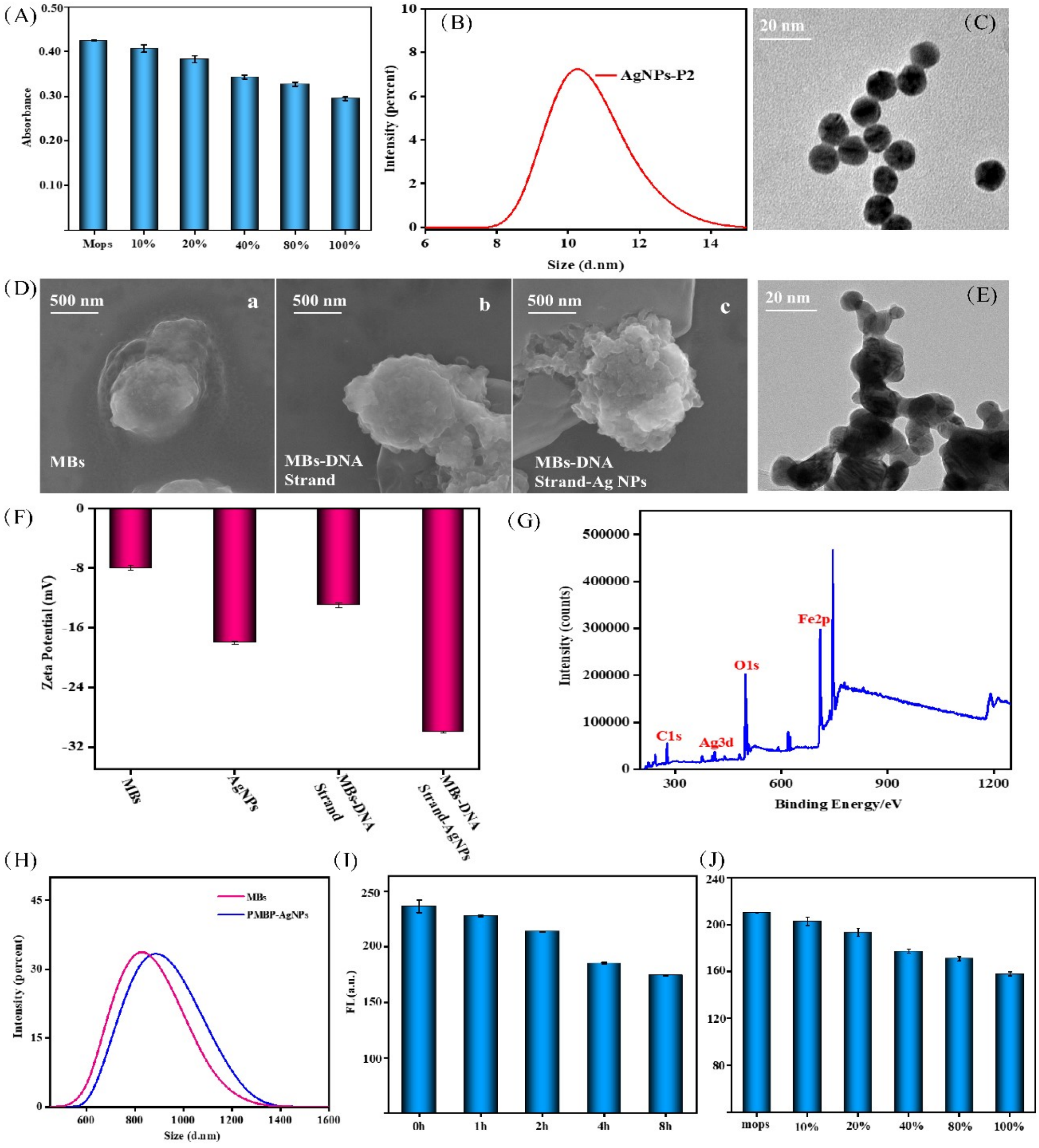

Figure S1. Characterization of silver nanomaterials and the PMBP probe. (A) Stability of silver nanomaterials in diluted human serum.(B) Dynamic light scattering (DLS) profile of P2 strand-modified silver nanomaterials.(C, E) Transmission electron microscopy (TEM) images of Ag NPs-P2 complexes before (C) and after (E) MTX addition, respectively.(D) TEM image of the intact PMBP probe (magnetic bead-P1-P2-Ag NPs complex).(F) Zeta potential profile of the intact PMBP probe.(G) X-ray photoelectron spectroscopy (XPS) spectrum of the intact PMBP probe.(H) DLS profile of the intact PMBP probe.(I) Time stability of the intact PMBP probe.(J) Stability of the intact PMBP probe in in diluted human serum. Data represent means ± SD. (n=5)

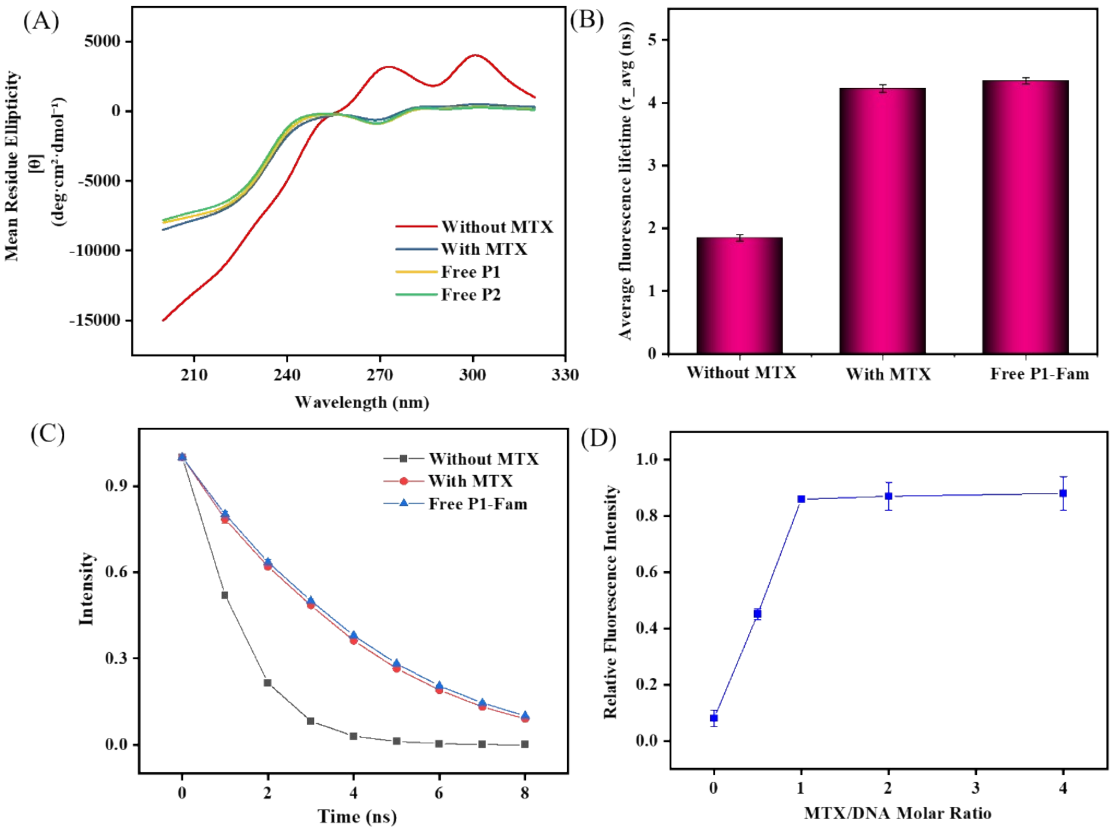

Figure S2. Validation of the FRET process. (A) Circular dichroism (CD) spectra of DNA strands before and after MTX binding. (B, C) Fluorescence lifetime measurements for DMSP process. (D) Optimization of the molar ratio between aptamer P1 and MTX. Data represent means ± SD. (n=4)

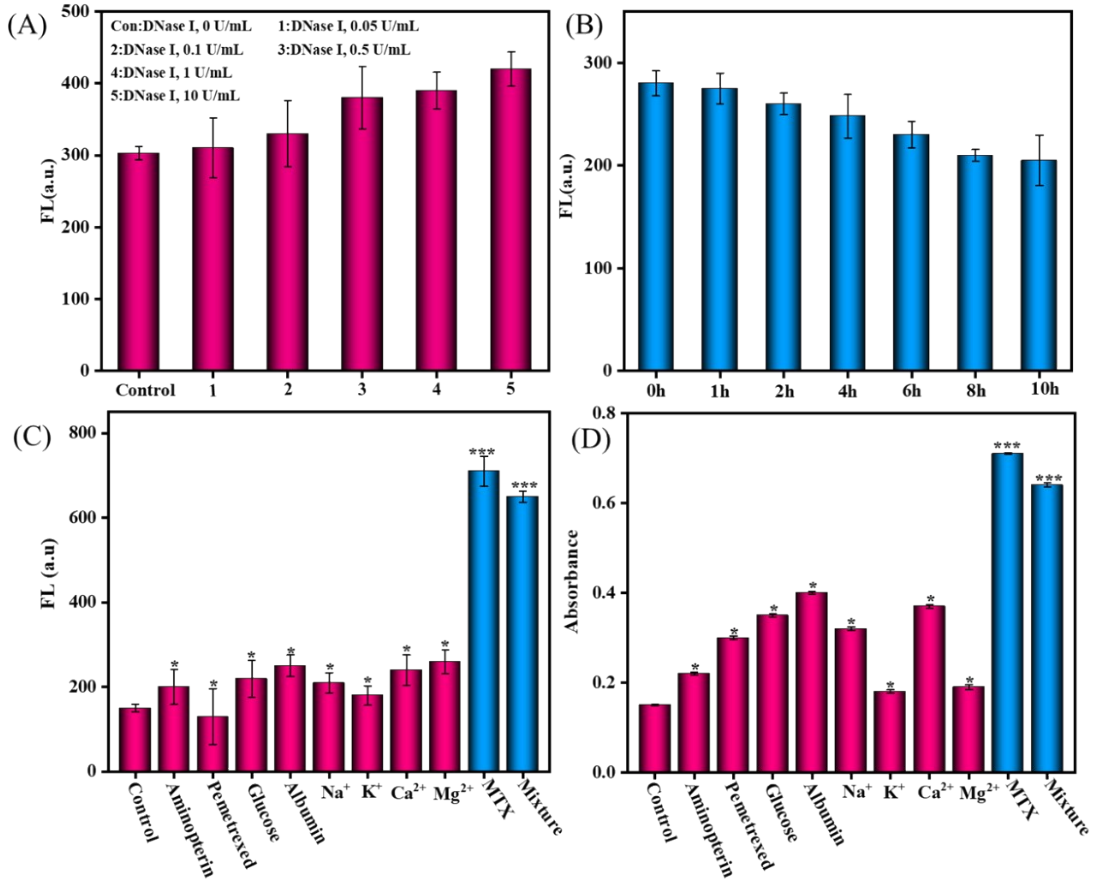

Figure S3. Biological evaluation of the PMBP probe. (A) Enzyme resistance of the probe at different enzyme concentrations. (B) Enzyme resistance of the probe over different incubation time periods. (C) Competitive binding assay of the probe with MTX structural analogs in diluted human serum via the fluorescence sensing mode. (D) Competitive binding assay of the probe with MTX structural analogs in diluted human serum via the UV-Vis sensing mode. ***$P \leq 0.001$ (unpaired t-test). Data represent means ± SD. Data represent means ± SD. (n=3)

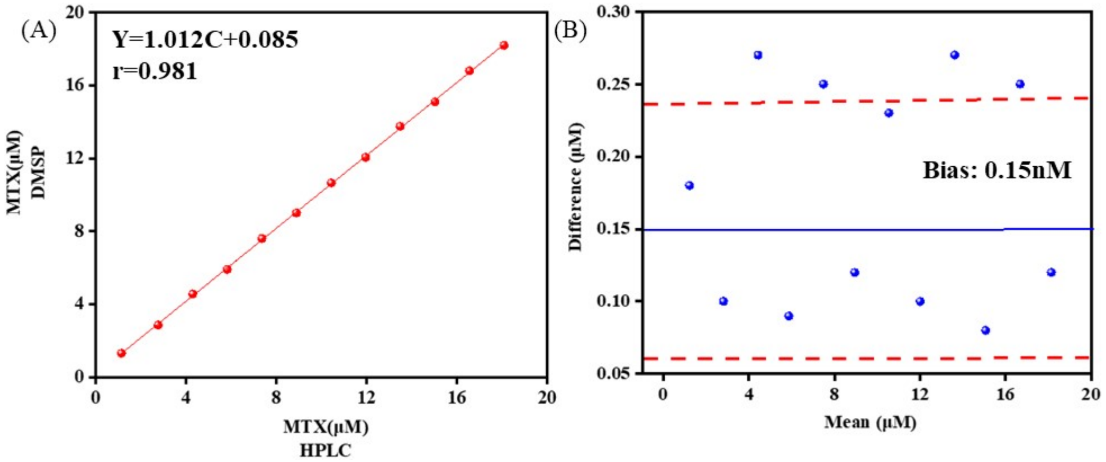

Figure S4. Comparative analysis of detection accuracy. (A, B) Correlation analysis results showing the consistency between the proposed DMSP method and the gold standard HPLC method.

Table S2 Recovery of MTX in human serum using the DMSP via fluorescence sensing

Spiked (μM)	Found (μM)	Recovery (%)	RSD (%)
0.50	0.57	114.01	2.66
4.00	3.94	98.50	1.81
8.00	8.15	101.87	2.21
20.00	21.05	105.24	0.43

Table S3 Precision of MTX in human serum using the DMSP via fluorescence sensing

Spiked (μM)	Intra-day Variability (μM)	Intra-day RSD (%)	Inter-day Variability (μM)	Inter-day RSD (%)	Inter-operator Variability (μM)	Inter-operator RSD (%)
0.50	0.58	3.11	0.53	3.25	0.52	3.85
4.00	3.91	2.30	3.95	3.15	3.81	4.26
8.00	8.19	1.20	8.05	3.11	7.98	3.23
20.00	21.32	2.45	21.15	3.25	20.18	4.11

Table S4 Precision of MTX in human serum using the HPLC via fluorescence sensing

Spiked (μM)	Intra-day Variability (μM)	Intra-day RSD (%)	Inter-day Variability (μM)	Inter-day RSD (%)	Inter-operator Variability (μM)	Inter-operator RSD (%)
0.50	0.56	2.80	0.57	4.20	0.55	4.85
4.00	3.92	2.30	3.94	3.05	3.89	3.85
8.00	8.12	2.20	8.15	3.07	8.08	4.22
20.00	21.02	2.45	21.05	3.55	20.98	4.50

Table S5 Recovery of MTX in human serum using the DMSP via UV sensing

Spiked (μM)	Found (μM)	Recovery (%)	RSD (%)
0.50	0.49	98.03	0.76
8.00	8.31	103.85	1.81
20.00	20.58	102.92	0.62

Table S6 Precision of MTX in human serum using the DMSP via UV sensing

Spiked (μM)	Intra-day Variability (μM)	Intra-day RSD (%)	Inter-day Variability (μM)	Inter-day RSD (%)	Inter-operator Variability (μM)	Inter-operator RSD (%)
0.50	0.43	3.33	0.56	4.53	0.6	5.85
8.00	8.98	3.45	8.47	5.05	8.49	5.57
20.00	20.78	3.25	21.33	4.79	21.08	4.99

Table S7 Precision of MTX in human serum using the HPLC via UV sensing

Spiked (μM)	Intra-day Variability (μM)	Intra-day RSD (%)	Inter-day Variability (μM)	Inter-day RSD (%)	Inter-operator Variability (μM)	Inter-operator RSD (%)
0.50	0.48	3.10	0.53	4.33	0.59	4.85
8.00	8.28	3.30	8.94	5.05	8.89	5.45
20.00	20.55	4.20	21.05	4.77	20.78	4.92

Reference

(1) Beyene, H. D.; Werkneh, A. A.; Bezabh, H. K.; et al. Synthesis paradigm and applications of silver nanoparticles (AgNPs), a review. *Sustain. Mater. Technol*. 2017, 13, 18-23.

(2) He, J.; Wang, J.; Zhang, M.; et al. Selection of a structure-switching aptamer for the specific methotrexate detection. *ACS Sens*. 2021, 6 (6), 2436-2441.
